# The first complete mitochondrial genome of sesame (*Sesamum indicum* L.)

**DOI:** 10.1590/1678-4685-GMB-2024-0064

**Published:** 2024-12-02

**Authors:** Mingcheng Wang, Rui Li, Xuchen Yang

**Affiliations:** 1 Chengdu University, Institute for Advanced Study, Chengdu, China.; 2 Engineering Research Center of Sichuan-Tibet Traditional Medicinal Plant, Chengdu, China.; 3 Guangzhou University, School of Life Sciences, Innovative Center of Molecular Genetics and Evolution, Guangdong Provincial Key Laboratory of Plant Adaptation and Molecular Design, Guangzhou, China.; 4 Guangzhou University, School of Life Sciences, Innovative Center of Molecular Genetics and Evolution, Guangzhou Key Laboratory of Crop Gene Editing, Guangzhou, China.

**Keywords:** Sesame, mitogenome assembly and annotation, multi-chromosomal mitogenome structure, PacBio high-fidelity sequencing

## Abstract

Sesame (*Sesamum indicum* L.), an important oilseed crop, has garnered considerable interest. The nuclear and chloroplast genomes of sesame have been extensively applied to sesame genetics and genomics research. The mitochondrial (mt) genome of sesame, however, has not been sequenced and annotated. In order to solve this issue, we reconstructed the first mt genome of sesame using third-generation sequencing data. The sesame mt genome was 724,998 bp in size and had 22 circular chromosomes. A total of 66 genes were annotated, including 37 protein-coding genes, 26 transfer RNAs, and three ribosomal RNAs. We investigated the codon usage patterns, simple sequence repeats, long tandem repeats, and dispersed repeats of the sesame mt genome. Furthermore, we investigated the DNA transfer from chloroplast to mitochondrion and compared the sesame mt genome to two other Lamiales mt genomes. Given the economic importance of this crop, our presented sesame mt genome is a valuable genomic resource and will allow for more comprehensive studies on sesame and related species.

Almost all higher plants have three distinct genomes in their cells: the nuclear, chloroplast (cp), and mitochondrial (mt) genomes (Leon et al., 1998). The latter two, referred to as organelle genomes (Wang J et al., 2024), are much smaller and contain less genetic information than the nuclear genome. However, the genetic information necessary for plant growth, development, and inheritance is found in the organelle genomes (Budar and Roux, 2011; Perico and Sparkes, 2018; Lee and Kang, 2020). The cp genomes, which are closely tied to the photosynthesis process, have highly conserved genome size, structure, and gene content (Daniell et al., 2016). In contrast, there are notable variations in the size and structure of mt genomes among different plant species, which might be due to repeat-driven chromosome recombination and foreign DNA insertion (Wu et al., 2022). Reconstructing a complete plant mt genome has been challenging due to its intricate structure (Wang J *et al.*, 2024). As a result, plant mt genomes have been far less studied than cp genomes, despite their considerable potential in plant genetics and genomics studies. Sesame (*Sesamum indicum* L.), a major oilseed crop, also exhibits this study bias. Recently, second- and third-generation sequencing has allowed for extensive analysis of the sesame’s nuclear and cp genomes (Yi and Kim, 2012; Wang L *et al.*, 2014; Wang M *et al.*, 2022). The sesame’s mt genome, however, has not been sequenced and annotated, which prevents comprehensive genetic and genomic research from being done on this plant. The availability of a complete sesame mt genome is crucial for understanding its genetic diversity, evolutionary history, and potential for crop improvement. To address this problem, we used third-generation sequencing data to reconstruct the sesame mt genome. In addition, we annotated this mt genome and investigated its genomic characteristics. By making the complete sesame mt genome available, we aim to provide a valuable resource for future studies in plant genetics, genomics, and breeding.

The PacBio high-fidelity (HiFi) sesame sequencing data, previously used to assemble the nuclear genome (Wang M et al., 2022), were acquired from the NCBI database under the BioProject PRJNA875260. There were 2,011,950 HiFi long reads, totaling 34.33 Gb in size, with a reads N50 value of 16.96 kb. PMAT (Bi *et al.*, 2024), a recently developed mitogenome assembler, was used to *de novo* assemble the sesame mt genome based on these HiFi reads using the main parameters “autoMito -st hifi -g 317M -fc 0.4”. Subsequently, potential erroneous links and forks were eliminated manually by examining the read depths of the connected contigs in Bandage (Wick et al., 2015). After these steps, the complete sesame mt genome was obtained. The IPMGA online tool (http://www.1kmpg.cn/ipmga/) was used to annotate the protein-coding, transfer RNA (tRNA), and ribosomal RNA (rRNA) genes in the mt genome. RNA editing sites were identified using Deepred-Mt (Edera et al., 2021), and only predictions with probability values exceeding 0.9 were deemed reliable. CodonW v1.4.2 (Peden, 1999) was used to compute the relative synonymous codon usage (RSCU) values in order to analyze the codon usage patterns of the protein-coding genes (PCGs). The distribution of the genes within the mt genome was visualized using the web tool OGDRAW v1.3.1 (Greiner et al., 2019; https://chlorobox.mpimp-golm.mpg.de/OGDraw.html). The online MISA server (Beier et al., 2017; https://webblast.ipk-gatersleben.de/misa/) was used to detect simple sequence repeats (SSRs) with a minimum number of repetitions set to 10 for mononucleotide repeats, five for dinucleotide repeats, four for trinucleotide repeats, and three for tetra-, penta-, and hexanucleotide repeats. Tandem Repeats Finder’s online version (Benson, 1999; https://tandem.bu.edu/trf/home) was used to identify tandem repeat sequences using the default settings. REPuter’s online version (Kurtz et al., 2001; https://bibiserv.cebitec.uni-bielefeld.de/reputer) was used to search for dispersed repeats, with the parameters of minimum repeat size and hamming distance set to 30 and 3 bp, respectively. To investigate DNA transfer from chloroplast to mitochondrion, we downloaded the sesame cp genome (GenBank accession number: NC_016433.2) from NCBI and used BLASTN v2.2.31+ (Camacho et al., 2009) with the main settings “-evalue 1e-5 -gapopen 5 -word_size 9” to find homologous fragments between the cp and mt genomes. The sesame mt genome was also compared to the mt genomes of two other Lamiales species, *Utricularia reniformis* (GenBank accession number: NC_034982.1; Silva et al., 2017) and *Salvia splendens* (GenBank accession number: PNBA02000024.1), using BLASTN.

The complete sesame mt genome exhibited a multi-chromosomal structure, comprising 22 circular chromosomes totaling 724,998 bp with an overall GC content of 44.33% (Figure 1 and Table S1). The longest chromosome was 181,805 bp long, and the shortest was only 17 bp long. The sesame mt genome showed an average coverage depth of 729× from HiFi reads, with 20 chromosomes having an average coverage depth exceeding 600×. Furthermore, PMAT software successfully mapped 436 out of 544 conserved plant mt genes to the sesame mt genome, demonstrating its high level of completeness. We identified 66 genes on 11 chromosomes, comprising 37 PCGs, 26 tRNAs, and three rRNAs (Table 1). The coding regions of these genes made up 5.35% of the entire mt genome (Table S2). The rRNAs had the highest GC content (51.32%), followed by the tRNAs (50.41%) and PCGs (42.52%). One PCG (*sdh3*) and three tRNAs (*trnD-GUC*, *trnE-UUC*, and *trnM-CAU*) were found to have duplicated copies. There were 11 genes with introns: six (*ccmFC*, *cox1*, *cox2*, *rps10*, *rps3*, and *trnI-AAU*) had one intron, three (*nad1*, *nad4*, and *nad7*) had three introns, and two (*nad2* and *nad5*) had four. Additionally, three genes (*cox2*, *nad1*, and *nad5*) were found to be trans-chromosomal (Table S3).

**Figure 1-:**
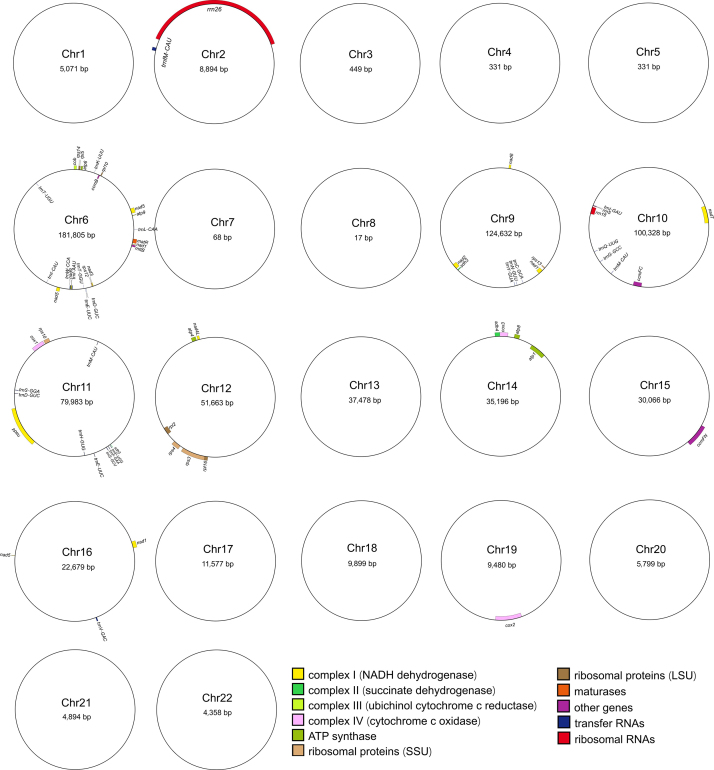
Circular maps of the 22 chromosomes of the sesame mitochondrial genome**.** Genes from the different functional groups are denoted in different colors.

**Table 1-:** Gene annotation for the sesame mitochondrial genome. The values within parentheses after the gene names are the copy numbers of duplicated genes. The asterisks after gene names reflect the intron numbers of the genes.

Gene groups	Gene names
Subunit of ATPase	*atp1*, *atp4*, *atp6*, *atp8*, *atp9*
Cytochrome c biogenesis	*ccmB*, *ccmC*, ccmFC*, *ccmFN*
Apocytochrome b	*cob*
Subunit of cytochrome c oxidase	cox1*, cox2*, *cox3*
Maturase R	*matR*
Transport membrane protein	*mttB*
Subunit of NADH dehydrogenase	nad1***, nad2****, *nad3*, nad4***, *nad4L*, nad5****, *nad6*, nad7***, *nad9*
Small subunit of ribosome	rps10*, *rps12*, *rps13*, *rps14*, rps3*, *rps4*
Large subunit of ribosome	*rpl10*, *rpl16*, *rpl2*, *rpl5*
Subunit of succinate dehydrogenase	*sdh3* (2), s*dh4*
Ribosomal RNAs	*rrn18*, *rrn26*, *rrn5*
Transfer RNAs	trnI-AAU*, *trnI-GAU*, *trnL-CAA*, *trnT-UGU*, *trnC-GCA*, *trnD-GUC* (2), *trnE-UUC* (2), *trnF-GAA*, *trnG-GCC*, *trnH-GUG*, *trnK-UUU*, *trnM-CAU* (2), *trnfM-CAU*, *trnI-CAU*, *trnN-GUU*, *trnP-UGG*, *trnQ-UUG*, *trnS-GCU*, *trnS-GGA*, *trnT-GGU*, *trnV-GAC*, *trnW-CCA*, *trnY-GUA*

The 37 PCGs in the sesame mt genome encoded a total of 10,466 codons. Leucine was the most commonly used amino acid (1,113 codons; 10.63%), while cysteine was the least used (152; 1.45%). The majority of the PCGs (32 PCGs; 86.49%) used ATG as the start codon, with the exception of ACG in *nad4L*, *rps10*, and *rps4*, GTG in *rpl16*, and TTC in one copy of *sdh3*. Furthermore, TAA, TAG, and TGA were the stop codons in 35 (94.59%) of the PCGs, with corresponding usage rates of 40.54%, 18.92%, and 35.14%, respectively. Two unusual stop codons, CAA and CGA, were identified in *atp6* and *rps10*, respectively. The unusual start and stop codons might be due to RNA editing alterations. To get a better understanding of the RNA editing events in the sesame mt genome, we predicted 467 high-confidence C to U RNA editing sites in the coding regions of 35 PCGs (Table S4). There were more than 30 RNA editing sites in four genes (*ccmB*, *nad4*, *ccmC*, and *mttB*), but only two in five genes (*atp9*, *rpl10*, *rpl2*, *rps14*, and *sdh3*) and none in two genes (*atp1* and one copy of *sdh3*). Interestingly, RNA editing events were more likely to occur in the first and second codon positions than in the third position, accounting for 32.98%, 62.53%, and 4.50% of the total RNA editing sites across the three positions, respectively (Table S4). Codon usage pattern analysis revealed that 29 codon types had RSCU values > 1, with 27 of them ending in either A or T (Figure S1). Additionally, there were 30 T and 26 A nucleotides in the 87 bases that the 29 codons encoded. These results indicate a strong AT bias in the encoded codons of the sesame mt genome, and this bias was also frequently observed in other higher plants (Qu et al., 2023). Furthermore, two codons (ATG and TGG) displayed no codon usage bias (RSCU = 1), while 31 codons had RSCU values < 1.

There were 187 SSRs in the sesame mt genome (Table S5), with tetranucleotide repeats being the most common (85 SSRs; 45.45%). This was followed by 37 (19.79%) dinucleotide, 36 (19.25%) mononucleotide, 22 (11.76%) trinucleotide, 6 (3.21%) pentanucleotide, and 1 (0.53%) hexanucleotide repeats. In particular, A/T bases made up the majority (88.89%) of the monomeric repeats, and the overall AT content of all SSR sequences was 73.14%. We identified nine long tandem repeats with lengths of 26 to 106 bp (Table S6), seven of which had match scores > 80%, and all of which were present in multiple copies. Aside from the SSRs and tandem repeats, 1,226 pairs of dispersed repeats totaling 98,526 bp were identified (Table S7), including 628 pairs of palindromic repeats and 598 pairs of forward repeats, but no complementary or reverse repeats were detected. The longest forward and palindromic repeats were 331 and 335 bp long, respectively. The majority of the dispersed repeats (1,196 repeats; 85.15%) were less than 50 bp in length, with only 15 repeats exceeding 100 bp.

A total of 76 fragments were recognized as DNA that had transferred from the cp to the mt genome, totaling 51,135 bp and accounting for 7.05% of the sesame mt genome (Figure S2). Twelve fragments were longer than 1,000 bp, with the longest fragment measuring 8,080 bp. These fragments revealed 11 complete tRNAs (*trnD-GUC*, *trnE-UUC*, *trnH-GUG*, *trnI-GAU*, *trnL-CAA*, *trnM-CAU*, *trnN-GUU*, *trnS-GGA*, *trnT-GGU*, *trnV-GAC*, and *trnW-CCA*), and one partial tRNA (*trnI-AAU*). A comparative analysis of the mt genomes of *S. splendens* and sesame revealed 162 homologous blocks totaling 135,384 bp, 42 of which were longer than 1,000 bp (Figure S3A). When the mt genomes of *U. reniformis* and sesame were compared, many more homologous blocks (346) totaling 157,269 bp were found, 37 of which were longer than 1,000 bp (Figure S3B). The sesame mt genome had low collinearity with the other two Lamiales mt genomes, and this could be due to frequent chromosome breakage and recombination events after their species diverged (Wynn and Christensen, 2019).

In this study, we introduced the first fully sequenced and annotated sesame mt genome. Unlike other released Lamiales single-chromosomal mt genomes, our reported sesame mt genome comprised a large number of circular chromosomes and had little collinearity with other mt genomes. These observations provide valuable insights into the evolutionary history of mitochondrial structure and functional genes among Lamiales species, but more novel mt genomes within this order will be required to fully understand the karyotype evolution of Lamiales. Furthermore, our high-quality gene annotations and repeat sequence database predicted from our assembled sesame mt genome will be useful for genetically improving, identifying cultivars in, and doing comparative genomics studies on this economically important crop and its related species. Notably, the sesame mt genome adds valuable insights given the shortage of sesame genetic resources by enabling us to better understand and make use of this species in the future.

## Supplementary material

The following online material is available for this article:

Figure S1 - Distribution of RSCU values of codons encoded by protein-coding genes in the sesame mitochondrial genome.

Figure S2 - The DNA fragments that are shared between the chloroplast and mitochondrial genomes of sesame.

Figure S3 - The DNA fragments that are shared between the mitochondrial genomes of sesame and two other Lamiales species, *Salvia splendens* and *Utricularia reniformis*.

Table S1 - Summary of 22 chromosomes of the sesame mitochondrial genome.

Table S2 - Summary of coding and non-coding regions in the sesame mitochondrial genome.

Table S3 - Summary of trans-chromosomal genes in the sesame mitochondrial genome.

Table S4 - Summary of C to U RNA editing sites in the sesame mitochondrial genome.

Table S5 - Summary of SSRs in the sesame mitochondrial genome.

Table S6 - Summary of long tandem repeats in the sesame mitochondrial genome.

Table S7 - Summary of dispersed repeats in the sesame mitochondrial genome.

## References

[B1] Beier S, Thiel T, Münch T, Scholz U, Mascher M (2017). MISA-web: A web server for microsatellite prediction. Bioinformatics.

[B2] Benson G (1999). Tandem repeats finder: A program to analyze DNA sequences. Nucleic Acids Res.

[B3] Bi C, Shen F, Han F, Qu Y, Hou J, Xu K, Xu L, He W, Wu Z, Yin T (2024). PMAT: An efficient plant mitogenome assembly toolkit using low coverage HiFi sequencing data. Hortic Res.

[B4] Budar F, Roux F (2011). The role of organelle genomes in plant adaptation: time to get to work!. Plant Signal Behav.

[B5] Camacho C, Coulouris G, Avagyan V, Ma N, Papadopoulos J, Bealer K, Madden TL (2009). BLAST+: architecture and applications. BMC Bioinformatics.

[B6] Daniell H, Lin CS, Yu M, Chang WJ (2016). Chloroplast genomes: Diversity, evolution, and applications in genetic engineering. Genome Biol.

[B7] Edera AA, Small I, Milone DH, Sanchez-Puerta MV (2021). Deepred-Mt: Deep representation learning for predicting C-to-U RNA editing in plant mitochondria. Comput Biol Med.

[B8] Greiner S, Lehwark P, Bock R (2019). OrganellarGenomeDRAW (OGDRAW) version 1.3.1: Expanded toolkit for the graphical visualization of organellar genomes. Nucleic Acids Res.

[B9] Kurtz S, Choudhuri JV, Ohlebusch E, Schleiermacher C, Stoye J, Giegerich R (2001). REPuter: The manifold applications of repeat analysis on a genomic scale. Nucleic Acids Res.

[B10] Lee K, Kang H (2020). Roles of organellar RNA-binding proteins in plant growth, development, and abiotic stress responses. Int J Mol Sci.

[B11] Leon P, Arroyo A, Mackenzie S (1998). Nuclear control of plastid and mitochondrial development in higher plants. Annu Rev Plant Physiol Plant Mol Biol.

[B12] Peden JF (1999). Analysis of codon usage.

[B13] Perico C, Sparkes I (2018). Plant organelle dynamics: Cytoskeletal control and membrane contact sites. New Phytol.

[B14] Qu Y, Zhou P, Tong C, Bi C, Xu LA (2023). Assembly and analysis of the Populus deltoides mitochondrial genome: The first report of a multicircular mitochondrial conformation for the genus Populus. J For Res.

[B15] Silva SR, Alvarenga DO, Aranguren Y, Penha HA, Fernandes CC, Pinheiro DG, Oliveira MT, Michael TP, Miranda VFO, Varani AM (2017). The mitochondrial genome of the terrestrial carnivorous plant Utricularia reniformis (Lentibulariaceae): Structure, comparative analysis and evolutionary landmarks. PLoS One.

[B16] Wang J, Kan S, Liao X, Zhou J, Tembrock LR, Daniell H, Jin S, Wu Z (2024). Plant organellar genomes: Much done, much more to do. Trends Plant Sci.

[B17] Wang L, Yu S, Tong C, Zhao Y, Liu Y, Song C, Zhang Y, Zhang X, Wang Y, Hua W (2014). Genome sequencing of the high oil crop sesame provides insight into oil biosynthesis. Genome Biol.

[B18] Wang M, Huang J, Liu S, Liu X, Li R, Luo J, Fu Z (2022). Improved assembly and annotation of the sesame genome. DNA Res.

[B19] Wick RR, Schultz MB, Zobel J, Holt KE (2015). Bandage: Interactive visualization of de novo genome assemblies. Bioinformatics.

[B20] Wu ZQ, Liao XZ, Zhang XN, Tembrock LR, Broz A (2022). Genomic architectural variation of plant mitochondria - A review of multichromosomal structuring. J Syst Evol.

[B21] Wynn EL, Christensen AC (2019). Repeats of unusual size in plant mitochondrial genomes: Identification, incidence and evolution. G3 (Bethesda).

[B22] Yi DK, Kim KJ (2012). Complete chloroplast genome sequences of important oilseed crop Sesamum indicum L. PloS One.

