# Supplementary material for: The first complete mitochondrial genome of sesame (Sesamum indicum L.)
Source: Genet Mol Biol. 2024 Dec 2;47(4):e20240064. doi: 10.1590/1678-4685-GMB-2024-0064 (PMC11613652; doi:10.1590/1678-4685-GMB-2024-0064)
Supplement: Figure S1 - [file 1415-4757-GMB-47-4-e20240064-s1.pdf]

*Genetics and Molecular Biology*

https://doi.org/10.1590/1678-4685-GMB-2024-0064

# Supplementary Material to "The first complete mitochondrial genome of sesame (*Sesamum indicum* L.)"

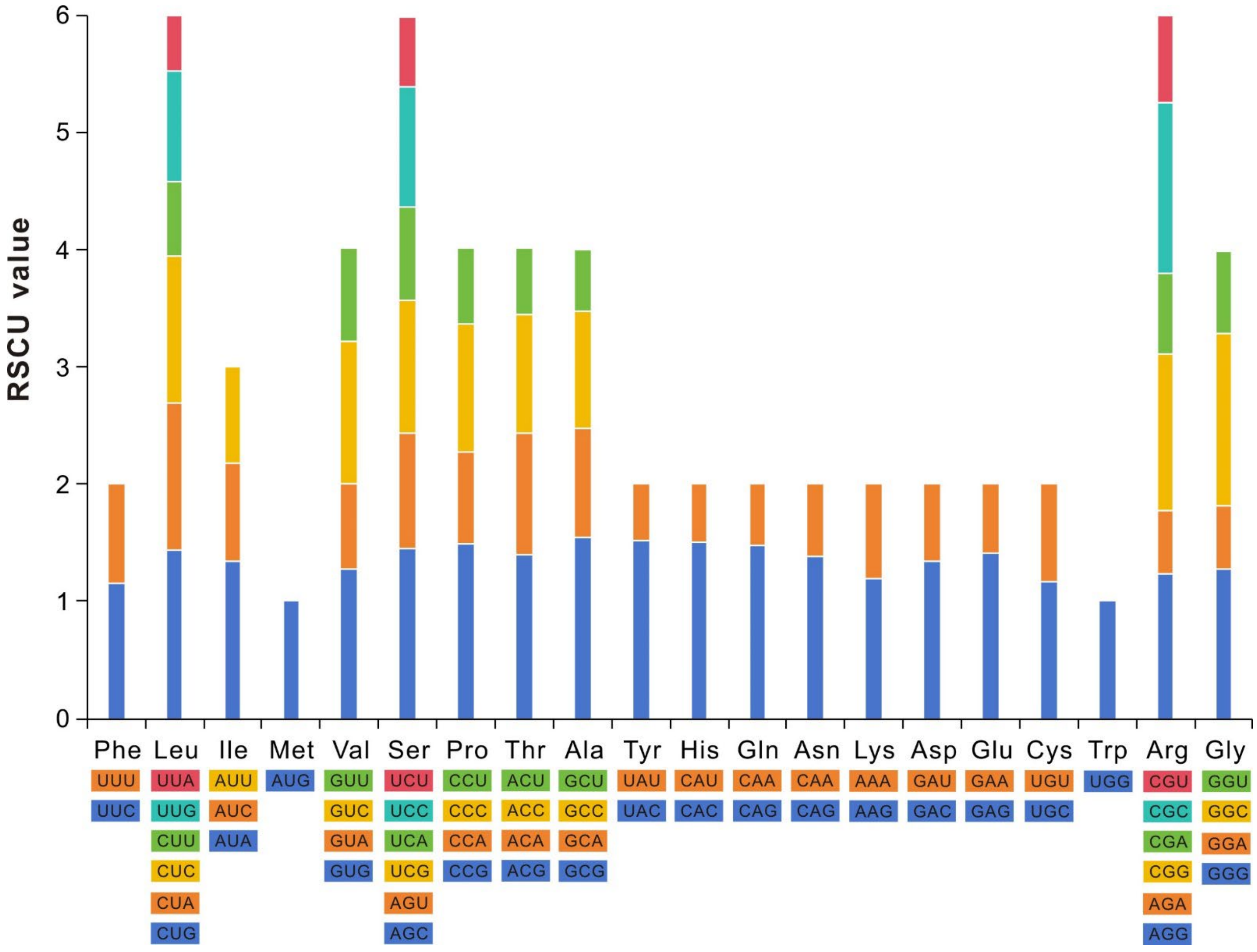


**Figure S1** - Distribution of RSCU values of codons encoded by protein-coding genes in the sesame mitochondrial genome.
