# Supplementary material for: The first complete mitochondrial genome of sesame (Sesamum indicum L.)
Source: Genet Mol Biol. 2024 Dec 2;47(4):e20240064. doi: 10.1590/1678-4685-GMB-2024-0064 (PMC11613652; doi:10.1590/1678-4685-GMB-2024-0064)
Supplement: Table S1 - [file 1415-4757-GMB-47-4-e20240064-s4.pdf]

Genetics and Molecular Biology
https://doi.org/10.1590/1678-4685-GMB-2024-0064

Supplementary Material to "The first complete mitochondrial genome of sesame (*Sesamum indicum* L.)"

Table S1 - Summary of 22 chromosomes of the sesame mitochondrial genome.

Chromosome	Size (bp)	Percent of mt genome (%)	GC content (%)	Coverage (×)
Chr1	5,071	0.70	45.81	698
Chr2	8,894	1.23	47.05	1,161
Chr3	449	0.06	44.77	1,341
Chr4	331	0.05	43.50	665
Chr5	331	0.05	43.50	665
Chr6	181,805	25.08	43.96	704
Chr7	68	0.01	47.06	2,190
Chr8	17	0.00	29.41	1,524
Chr9	124,632	17.19	44.63	700
Chr10	100,328	13.84	45.27	810
Chr11	79,983	11.03	44.42	725
Chr12	51,663	7.13	44.20	654
Chr13	37,478	5.17	43.12	690
Chr14	35,196	4.85	43.66	726
Chr15	30,066	4.15	44.34	702
Chr16	22,679	3.13	43.56	585
Chr17	11,577	1.60	44.32	1,272
Chr18	9,899	1.37	44.35	620
Chr19	9,480	1.31	44.03	587
Chr20	5,799	0.80	44.65	659
Chr21	4,894	0.68	43.77	654
Chr22	4,358	0.60	43.21	1,356
**Total**	**724,998**	**-**	**44.33**	**729**
