# Supplementary material for: The first complete mitochondrial genome of sesame (Sesamum indicum L.)
Source: Genet Mol Biol. 2024 Dec 2;47(4):e20240064. doi: 10.1590/1678-4685-GMB-2024-0064 (PMC11613652; doi:10.1590/1678-4685-GMB-2024-0064)
Supplement: Table S2 - [file 1415-4757-GMB-47-4-e20240064-s5.pdf]

*Genetics and Molecular Biology*
https://doi.org/10.1590/1678-4685-GMB-2024-0064

# Supplementary Material to "The first complete mitochondrial genome of sesame (*Sesamum indicum* L.)"

**Table S2 -** Summary of coding and non-coding regions in the sesame mitochondrial genome.

| Region | Size (bp) | Percent of mt genome (%) | GC content (%) |
|---|---|---|---|
| Protein-coding genes | 31,398 | 4.33 | 42.52 |
| tRNA genes | 1,964 | 0.27 | 50.41 |
| rRNA genes | 5,454 | 0.75 | 51.32 |
| Non-coding regions | 686,181 | 94.65 | 44.34 |
