# Supplementary material for: The first complete mitochondrial genome of sesame (Sesamum indicum L.)
Source: Genet Mol Biol. 2024 Dec 2;47(4):e20240064. doi: 10.1590/1678-4685-GMB-2024-0064 (PMC11613652; doi:10.1590/1678-4685-GMB-2024-0064)
Supplement: Table S3 - [file 1415-4757-GMB-47-4-e20240064-s6.pdf]

Genetics and Molecular Biology
https://doi.org/10.1590/1678-4685-GMB-2024-0064

Supplementary Material to “The first complete mitochondrial genome of sesame (*Sesamum indicum* L.)”

Table S3 - Summary of trans-chromosomal genes in the sesame mitochondrial genome.

Gene names	Chromosome	Start position	End position
nad1	Chr16	815	1,119
	Chr9	108,919	108,837
	Chr9	107,585	107,394
	Chr6	174,596	174,787
nad5	Chr6	7,841	8,070
	Chr6	8921	10,136
	Chr16	10,978	10,999
	Chr6	128,082	128,476
	Chr6	129,489	129,638
cox2	Chr17	1	678
	Chr19	2,975	3,058
