# Supplementary material for: The first complete mitochondrial genome of sesame (Sesamum indicum L.)
Source: Genet Mol Biol. 2024 Dec 2;47(4):e20240064. doi: 10.1590/1678-4685-GMB-2024-0064 (PMC11613652; doi:10.1590/1678-4685-GMB-2024-0064)
Supplement: Table S4 - [file 1415-4757-GMB-47-4-e20240064-s7.pdf]

Genetics and Molecular Biology
https://doi.org/10.1590/1678-4685-GMB-2024-0064

Supplementary Material to "The first complete mitochondrial genome of sesame (*Sesamum indicum* L.)"

Table S4 - Summary of C to U RNA editing sites in the sesame mitochondrial genome.

Gene name	Number of RNA editing sites	Number of RNA editing sites (1st	Number of RNA editing sites (2nd	Number of RNA editing sites (3rd
atp4	12	2	10	0
atp6	18	5	12	1
atp8	3	1	1	1
atp9	2	0	1	1
ccmB	38	15	22	1
ccmC	32	12	18	2
ccmFC	14	8	6	0
ccmFN	27	12	14	1
cob	9	8	1	0
cox1	14	3	10	1
cox2	11	3	8	0
cox3	9	2	7	0
matR	10	2	7	1
mttB	32	19	12	1
nad1	16	7	7	2
nad2	26	8	17	1
nad3	15	4	11	0
nad4	37	9	27	1
nad4L	14	2	12	0
nad5	24	3	21	0
nad6	10	4	6	0
nad7	25	6	16	3
nad9	9	3	6	0
rpl10	2	0	2	0
rpl16	5	1	2	2
rpl2	2	0	2	0
rpl5	6	2	4	0
rps10	3	2	1	0
rps12	4	1	3	0
rps13	3	1	2	0

Gene name	Number of RNA editing sites	Number of RNA editing sites (1st	Number of RNA editing sites (2nd	Number of RNA editing sites (3rd
rps14	2	1	1	0
rps3	10	2	7	1
rps4	14	4	10	0
sdh3	2	1	1	0
sdh4	7	1	5	1
