# Supplementary material for: The first complete mitochondrial genome of sesame (Sesamum indicum L.)
Source: Genet Mol Biol. 2024 Dec 2;47(4):e20240064. doi: 10.1590/1678-4685-GMB-2024-0064 (PMC11613652; doi:10.1590/1678-4685-GMB-2024-0064)
Supplement: Table S5 - [file 1415-4757-GMB-47-4-e20240064-s8.pdf]

Genetics and Molecular Biology
https://doi.org/10.1590/1678-4685-GMB-2024-0064

Supplementary Material to "The first complete mitochondrial genome of sesame (*Sesamum indicum* L.)"

Table S5 - Summary of SSRs in the sesame mitochondrial genome.

**SSR type**	**Number of repeats**											**Total**
	3	4	5	6	7	8	9	10	11	12	13	**number**
Mononucleotide	0	0	0	0	0	0	0	27	6	2	1	36
Dinucleotide	0	0	28	8	0	0	1	0	0	0	0	37
Trinucleotide	0	20	2	0	0	0	0	0	0	0	0	22
Tetranucleotide	80	4	1	0	0	0	0	0	0	0	0	85
Pentanucleotide	5	1	0	0	0	0	0	0	0	0	0	6
Hexanucleotide	0	1	0	0	0	0	0	0	0	0	0	1
