# Supplementary material for: The first complete mitochondrial genome of sesame (Sesamum indicum L.)
Source: Genet Mol Biol. 2024 Dec 2;47(4):e20240064. doi: 10.1590/1678-4685-GMB-2024-0064 (PMC11613652; doi:10.1590/1678-4685-GMB-2024-0064)
Supplement: Table S6 - [file 1415-4757-GMB-47-4-e20240064-s9.pdf]

*Genetics and Molecular Biology*
https://doi.org/10.1590/1678-4685-GMB-2024-0064

# Supplementary Material to “The first complete mitochondrial genome of sesame (*Sesamum indicum* L.)”

**Table S6 -** Summary of long tandem repeats in the sesame mitochondrial genome.

| **Position** | **Length (bp)** | **Period size** | **Copy number** | **Percent matches (%)** |
|---|---|---|---|---|
| Chr10: 8465--8502 | 38 | 20 | 1.9 | 88 |
| Chr12: 28376--28481 | 106 | 29 | 3.7 | 100 |
| Chr13: 8925--8971 | 47 | 24 | 2 | 86 |
| Chr15: 24760--24785 | 26 | 13 | 2 | 100 |
| Chr19: 7483--7535 | 53 | 21 | 2.5 | 79 |
| Chr19: 7464--7553 | 90 | 24 | 4.1 | 72 |
| Chr6: 84902--84941 | 40 | 18 | 2.3 | 86 |
| Chr6: 165924--165952 | 29 | 15 | 2 | 93 |
| Chr9: 93766--93801 | 36 | 18 | 1.9 | 88 |
