# Supplementary material for: The first complete mitochondrial genome of sesame (Sesamum indicum L.)
Source: Genet Mol Biol. 2024 Dec 2;47(4):e20240064. doi: 10.1590/1678-4685-GMB-2024-0064 (PMC11613652; doi:10.1590/1678-4685-GMB-2024-0064)
Supplement: Table S7 - [file 1415-4757-GMB-47-4-e20240064-s10.pdf]

Genetics and Molecular Biology
https://doi.org/10.1590/1678-4685-GMB-2024-0064

Supplementary Material to "The first complete mitochondrial genome of sesame (*Sesamum indicum* L.)"

Table S7 - Summary of dispersed repeats in the sesame mitochondrial genome.

Length (bp)	# of palindromic repeats	# of forward repeats
< 40	418	416
[40,49]	117	93
[50,59]	41	43
[60,69]	25	18
[70,79]	12	13
[80,89]	8	2
[90,99]	1	4
>= 100	6	9
